# Clinical features, comorbidities, complications and treatment options in severe and non‐severe COVID‐19 patients: A systemic review and meta‐analysis

**DOI:** 10.1002/nop2.718

**Published:** 2020-11-27

**Authors:** Mohan Giri, Anju Puri, Ting Wang, Shuliang Guo

**Affiliations:** ^1^ Department of Respiratory and Critical Care Medicine The First Affiliated Hospital of Chongqing Medical University Chongqing China

**Keywords:** clinical features, comorbidities, complications, COVID‐19, nurses, nursing, treatment

## Abstract

**Objectives:**

The aim of this analysis was to assess the prevalence of clinical features, comorbidities, complications and treatment options in the patients with COVID‐19 and compare incidence of these clinical data in severe and non‐severe patients.

**Design:**

Systemic review and Meta‐analysis.

**Methods:**

PubMed, Embase, Scopus and Web of Sciences databases were searched to identify relevant papers until 20 July 2020. All studies comparing clinical data of severe and non‐severe patients of COVID‐19 were included. Heterogeneity across included studies was determined using Cochrane's *Q* test and the *I* ^2^ statistic. Results were expressed as odds ratio with accompanying 95% confidence intervals.

**Results:**

Twelve studies with 3,046 patients were included. The result showed the most prevalent clinical symptoms were fever 88.3%, cough 62.2%, fatigue 39.5% and dyspnoea 31.5%. Further meta‐analysis showed incidence of fever, cough, fatigue and dyspnoea was higher in severe patients. The most prevalent comorbidities were hypertension 22.6%, diabetes 11.5%, cardiovascular disease 10.3% and cancer 2.5%. We found that compared with non‐severe patients, the symptoms, existing comorbidities and complications are prevalent in severe COVID‐19 patients. Future well‐methodologically designed studies from other populations are strongly recommended.

## 1 INTRODUCTION

During the past two decades, the outbreak of the two betacoronaviruses, severe acute respiratory syndrome coronavirus (SARS‐CoV) and Middle East respiratory syndrome coronavirus (MERS‐CoV), have markedly affected humans, with mortality rates of 10% for SARS‐CoV and 35% for MERS‐CoV (Azhar et al., 2019; Hui & Zumla, 2019). Yet another outbreak, in the form of the coronavirus disease (COVID‐19) caused by SARS‐CoV‐2, which has been believed to be originated in Wuhan, China, at the end of 2019 possibly related to contact with a seafood market has rapidly spread in over 200 countries (Huang et al., 2020). The World Health Organization (WHO) declared the coronavirus outbreak a public health emergency of international concern on January 30 and pandemic on March 11. As of 16 August 2020, globally a total of 21,294,845 cases of the COVID‐19 have been reported, with death rate of around 3.6% (World Health Organization, n.d.).

SARS‐CoV‐2 shares several common important features with SARS‐CoV and MERS‐CoV causing epidemics with variable clinical severity featuring lower respiratory tract infections and extra‐respiratory manifestations. Unlike MERS‐CoV or SARS‐CoV infection, 2019‐novel coronavirus (2019‐nCoV) patients rarely developed intestinal signs and symptoms like diarrhoea (Assiri et al., 2013; Leung et al., 2003). Alike the clinical characteristics of SARS‐CoV and MERS‐CoV, most patients of COVID‐19 presented with fever, dry cough, dyspnoea, sore throat etc. (Guan, Ni et al., 2020; Huang et al., 2020; Wan et al., 2020; Wang, Hu et al., 2020).

Furthermore, the prevalence of hypertension, diabetes mellitus, cardiovascular disease and other comorbidities, as well as complications, also varied between the studies due to the various characteristics of the study populations (Guan, Ni et al., 2020; Huang et al., 2020; Wan et al., 2020; Wang, Hu et al., 2020; Zhang et al., 2020). Although there are many studies regarding the clinical characteristics and comorbidities, of COVID‐19, but there are limited studies that compared clinical characteristics, comorbidities, treatment options and complications of severe and non‐severe patients. The exponential growth of COVID‐19 cases is overwhelming healthcare systems of developing countries with limited sources. Currently, there are no proven vaccines or effective treatment against the virus. Therefore, healthcare workers assessment ability to distinguish between mild and severe COVID‐19 cases promptly could help save lives and boost healthcare system. The present systematic review and meta‐analysis were undertaken to provide a systemic evaluation and detailed estimate to draw the whole clinical picture of COVID‐19 in severe and non‐severe cases. This assessment will help frontline healthcare workers for emergency preparedness and response to SARS‐CoV‐2 and its severe outcomes. The main objectives of our meta‐analysis are as follows:

- To acquire more accurate conclusions on the clinical features, comorbidities, complications and treatment options among patients with COVID‐19.
- To compare clinical features comorbidities, complications and treatment options among severe and non‐severe patients.

## 2 METHODS

### 2.1 Eligibility criteria

The inclusion criteria were as follows: (a) study population: studies with patients diagnosed with COVID‐19; (b) comparative studies: studies that compare severe and non‐severe cases of COVID‐19; and (c) the studies reporting parameters of clinical features, comorbidities, complications and treatment. Non‐English studies, letters, case studies, editorials, conference abstracts, vaccination trials studies and articles with abstracts only were excluded. Studies with only paediatric cases were also excluded.

### 2.2 Information sources and Searching strategies

We conducted this systematic review and meta‐analysis according to the Preferred Reporting Items for Systematic Reviews and Meta‐Analyses (PRISMA) Statement. The PubMed, Embase, Scopus and Web of Sciences databases were searched for relevant papers. The last search was performed on 20 July 2020. The following search terms were used alone or in combination to find all relevant studies: ‘Novel coronavirus’, ‘Novel coronavirus 2019’, ‘Coronavirus Disease 2019’, ‘2019 nCoV’, ‘COVID‐19’, ‘SARS‐CoV‐2’, ‘novel coronavirus pneumonia’ and ‘severe acute respiratory syndrome coronavirus 2’, characteristics, clinical features, treatment, co‐morbidity, complications. The search was limited to articles published in English.

### 2.3 Data extraction and outcomes

All duplicate studies were excluded by using by EndNote X 8.0 software. The two investigators who performed the literature search also independently extracted the data from included studies. Disagreements were resolved with a third investigator. Microsoft Excel database was used to record all available information including variables: author, date, age, gender and number of participants in severe and non‐severe groups. The prevalence of clinical symptoms such as fever, cough, fatigue, dyspnoea, sore throat, headache, chest pain, comorbidities, complications and treatment options used including antiviral drugs, antibiotics, glucocorticoids, oxygen support, continuous renal replacement therapy (CRRT), non‐invasive ventilation (NIV) and invasive mechanical ventilation (IMV) was also recorded.

The primary outcome measure was to compare the prevalence of clinical feature, comorbidities, complications and treatment options in severe cases (ICU cases, patients with elevated TnT level, patients with cardiac injury, cases with SpO2 < 90% and patients with ARDS as the second choice if severe data were not provided) and non‐severe (non‐ICU cases, patients normal TnT level, patients without cardiac injury, cases with SpO2 ≥ 90% and patients without ARDS as the second choice if non‐severe data were not provided).

### 2.4 Risk of bias assessment

The potential risk of bias of the included studies was assessed using the MINORS, a methodological index for non‐randomized studies. The corresponding scores for comparative studies are 0–6, very low quality; 7–12, low quality; 13–18, moderate quality; and 19–24, high quality (Slim et al., 2003).

### 2.5 Statistical analysis of data

All statistical analyses were performed with OpenMeta Analyst version 10.10 (www.cebm.brown.edu/open_meta), a free open‐source program and RevMan software version 5.3. Meta‐analysis of proportions (and 95% CI) was calculated for the clinical symptoms, comorbidities, complications and treatment options. Binary random effect model was used as clinical data are varied across study population.

The prevalence of clinical symptoms, comorbidities, complications and treatments was illustrated with forest plots. With OR (Odds ratio) as the effect quantity, we used Mantel–Haenszel test with fixed or random effect for further meta‐analysis of the clinical symptoms, comorbidities, complications and treatments with statistical differences in severe and non‐severe patients. We evaluated heterogeneity across the studies by using the *I* ^2^ statistic and Cochran's *Q* test (Higgins et al., 2003). When *I* ^2^ < 50%, the fixed effect model was used, while random effect model was used when *I* ^2^ > 50%.

## 3 RESULTS

**Figure 1:**
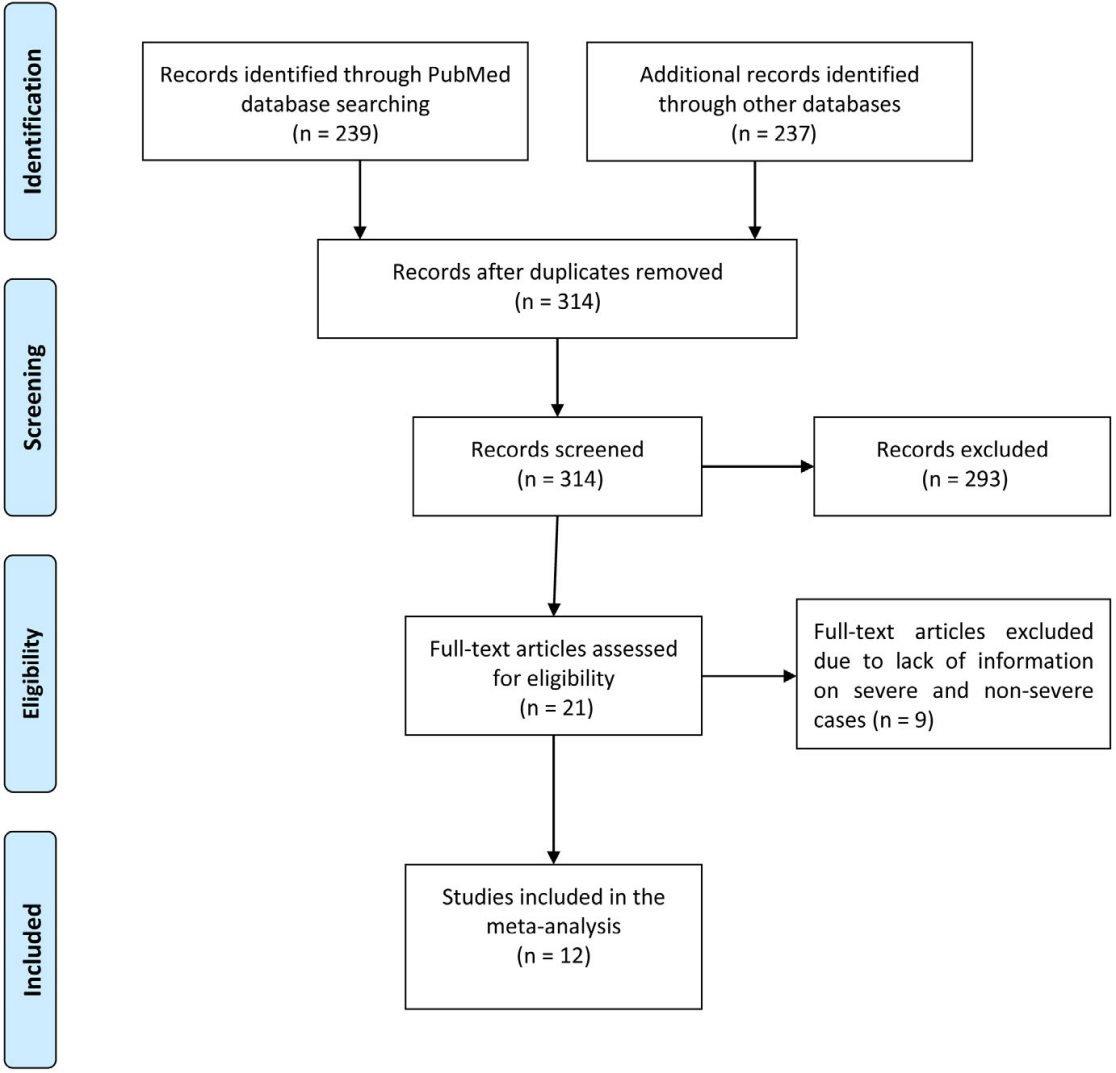
Flowchart of study selection process

A total of 476 papers were retrieved from the four databases, of which 162 studies were removed as duplicates. Remaining 314 studies were screened by title and abstract and, 293 studies were discarded according to exclusion criteria. After evaluating the 21 full texts, 9 studies were excluded due to presenting data that were irrelevant to our aim. Finally, 12 articles (Guan, Ni et al., 2020; Huang et al., 2020; Wan et al., 2020; Wang, Hu et al., 2020; Zhang et al., 2020), (Guo et al., 2020; Liu et al., 2020; Shi et al., 2020; Tian et al., 2020; Wang, Yang et al., 2020; Wu, Chen et al., 2020; Wu, Li et al., 2020) met the inclusion criteria but some of the required information of severe and non‐severe cases was not reported in all of the articles. A flow chart of study selection is shown in (Figure 1). All included studies were published in 2020 with different sample size that ranged from 41–1,099 patients. The risk of bias of eligible studies is presented in (Table 1). The 12 included studies scored between 18–21, with the mean overall score for all comparative studies being 19.6. According to the MINORS assessment, all studies were considered to have a low risk of bias for selection. The main characteristics of eligible studies are summarized in (Table 2).

**Table 1:** MINORS rating scale

Study	①	②	③	④	⑤	⑥	⑦	⑧	⑨	⑩	⑪	⑫	Score
Wang, Hu et al. (2020)	2	2	2	2	2	1	2	0	2	2	2	2	21
Wang, Yang et al. (2020)	2	2	2	2	2	1	2	0	2	2	2	2	21
Huang et al. (2020)	2	2	2	2	2	1	2	0	2	2	2	2	21
Wan et al. (2020)	2	2	2	2	2	1	1	0	2	2	2	2	20
Guan, Ni et al. ( 2020)	2	2	2	2	2	0	0	0	2	2	2	2	18
Zhang et al. (2020)	2	2	2	2	2	0	0	0	2	2	2	2	18
Tian et al. (2020)	2	2	2	2	2	1	1	0	2	2	2	2	20
Liu et al. (2020)	2	2	2	2	2	0	0	0	2	2	2	2	18
Wu, Chen et al. (2020)	2	2	2	2	2	1	1	0	2	2	2	2	20
Wu, Li et al. (2020)	2	2	2	2	2	1	0	0	2	2	2	2	19
Guo et al. (2020)	2	2	2	2	2	0	0	0	2	2	2	2	18
Shi et al. (2020)	2	2	2	2	2	1	2	0	2	2	2	2	21

Note

① A clearly stated aim; ② Inclusion of consecutive patients; ③ Prospective collection of data; ④ Endpoints appropriate to the aim of the study; ⑤ Unbiased assessment of the study endpoint; ⑥ Follow‐up period appropriate to the aim of the study; ⑦ Loss to follow‐up less than 5%; ⑧ Prospective calculation of the study size. ⑨ Appropriate selection of control group; ⑩ Synchronization of control group; ⑪ Baseline comparable between groups; ⑫ Appropriately statistical analysis. The global ideal score for comparative studies is 24.

**Table 2:** Characteristics of the included studies

Study (ref)	Country	Total patients	Age (years)	Male (%)	Severe (%)
Wang, Hu et al. (2020))	China	138	56	54.3	26.1
Wang, Yang et al. (2020))	China	69	42	46	20.3
Huang et al. (2020)	China	41	49	73	31.7
Wan et al. (2020)	China	135	47	53.3	29.6
(Guan, Ni et al. (2020)	China	1,099	47	58.1	15.7
Zhang et al. (2020)	China	140	57	50.7	41.4
Tian et al. (2020)	China	262	47.5	48.5	17.6
Liu et al. (2020)	China	78	38	50	14.1
Wu, Chen et al. (2020)	China	280	43.1	53.9	29.6
Wu, Li et al. (2020)	China	201	51	63.7	41.8
Guo et al. (2020)	China	187	58.5	48.7	27.8
Shi et al. (2020)	China	416	64	49.3	19.7

### 3.1 Demographic characteristics

The overall average age (±*SE*) of patients across 12 studies was 50 ± 2 years (range: 38–64 years). Men (53.8%, 95% CI: 50.3–57.3) were more likely to be infected than women counterparts. The proportion of severe patients in our study was 25.9% (95% CI: 20.8–31.0; Table 3). Chi‐square test showed that there was significant difference in gender between severe and non‐severe groups (*p* < .05).

**Table 3:** Meta‐analysis outcomes of clinical data (Random effect model)

Variable	Number of studies	Prevalence (%)	95% CI	*N*	*Q* ^a^	*I* ^2^ *_ _* ^b^	*T* ^2^_ _ ^c^	*p*
Male	12	53.8	50.3–57.3	1658	34.76	68	0.002	**<.001**
Female	12	46.2	42.7–49.7	1,385	34.76	68	0.002	**<.001**
Severe	12	25.9	20.8–31.0	692	111.56	90	0.007	**<.001**
Clinical features								
Fever	11	88.3	84.1–92.5	2,472	137.85	93	0.004	**<.001**
Cough	11	62.2	52.5–71.8	1746	279.67	96	0.025	**<.001**
Fatigue	10	39.5	28.2–50.8	954	371.79	98	0.031	**<.001**
Sore throat	6	11.7	6.0–17.4	250	90.93	95	0.005	**<.001**
Dyspnoea	10	31.5	22.3–40.7	729	284.10	97	0.020	**<.001**
Headache	8	11.1	6.1–16.1	275	129.27	95	0.005	**<.001**
Comorbidities								
Hypertension	10	22.6	16.7–28.5	513	93.19	90	0.007	**<.001**
Diabetes	10	11.5	9.0–14.1	254	26.55	66	0.001	.002
Cancer	10	2.5	1.3–3.6	61	31.22	71	0.000	**<.001**
COPD	9	1.6	0.8–2.4	42	16.59	52	0.000	.035
Cardiovascular disease	9	10.3	6.1–14.5	187	100.70	92	0.003	**<.001**
Chronic kidney disease	7	1.8	0.9–2.7	42	15.44	61	0.000	.017
Complications								
ARDS	7	22.2	10.6–33.8	324	268.47	98	0.023	**<.001**
Shock	5	1.3	0.1–2.5	29	15.76	75	0.000	.003
Acute kidney injury	6	3.6	1.4–5.8	45	31.77	84	0.000	**<.001**
Treatments								
Antiviral therapy	9	87.4	78.3–96.4	1775	1972.93	100	0.019	**<.001**
Antibiotic therapy	8	77.5	64.2–90.8	1605	991.20	99	0.036	**<.001**
Glucocorticoids	9	36.0	20.4–51.6	892	564.97	99	0.055	**<.001**
Oxygen support	7	67.6	52.0–83.1	1,201	308.80	98	0.042	**<.001**
CRRT	5	0.9	0.2–1.7	21	7.05	43	0.000	.133
NIV	7	24.4	10.7–38.0	404	458.17	99	0.033	**<.001**
IMV	6	8.4	4.1–12.6	164	133.34	96	0.003	**<.001**

Abbreviations: 95% CI, 95% confidence interval; ARDS, acute respiratory distress syndrome; COPD, chronic obstructive pulmonary disease; CRRT, continuous renal replacement therapy; IMV, invasive mechanical ventilation; NIV, non‐invasive ventilation.

*P*‐values < .05 were considered statistically significant.

^a^ Cochran's *Q* statistic for heterogeneity.

^b^ *I* ^2^ index to quantify the degree of heterogeneity.

^c^ Tau‐squared as a measure of heterogeneity.

### 3.2 Clinical features

The result of this meta‐analysis showed that the most prevalent clinical symptoms of COVID‐19 patients were fever (88.3%, 95% CI: 84.1%–92.5%), cough (62.2%, 95% CI: 52.5%–71.8%), fatigue (39.5%, 95% CI: 28.2%–50.8%), dyspnoea (31.5%, 95% CI: 22.3%–40.7%), sore throat (11.7%, 95% CI: 6.0%–17.4%) and headache (11.1%, 95% CI: 6.1–16.1; Table 3). In the estimate of clinical symptoms, significant heterogeneity (Cochran's *Q*) was observed among the identified studies (*p* < .001) with an *I* ^2^ varying from 93%–98% (Table 3).

**Figure 2:**
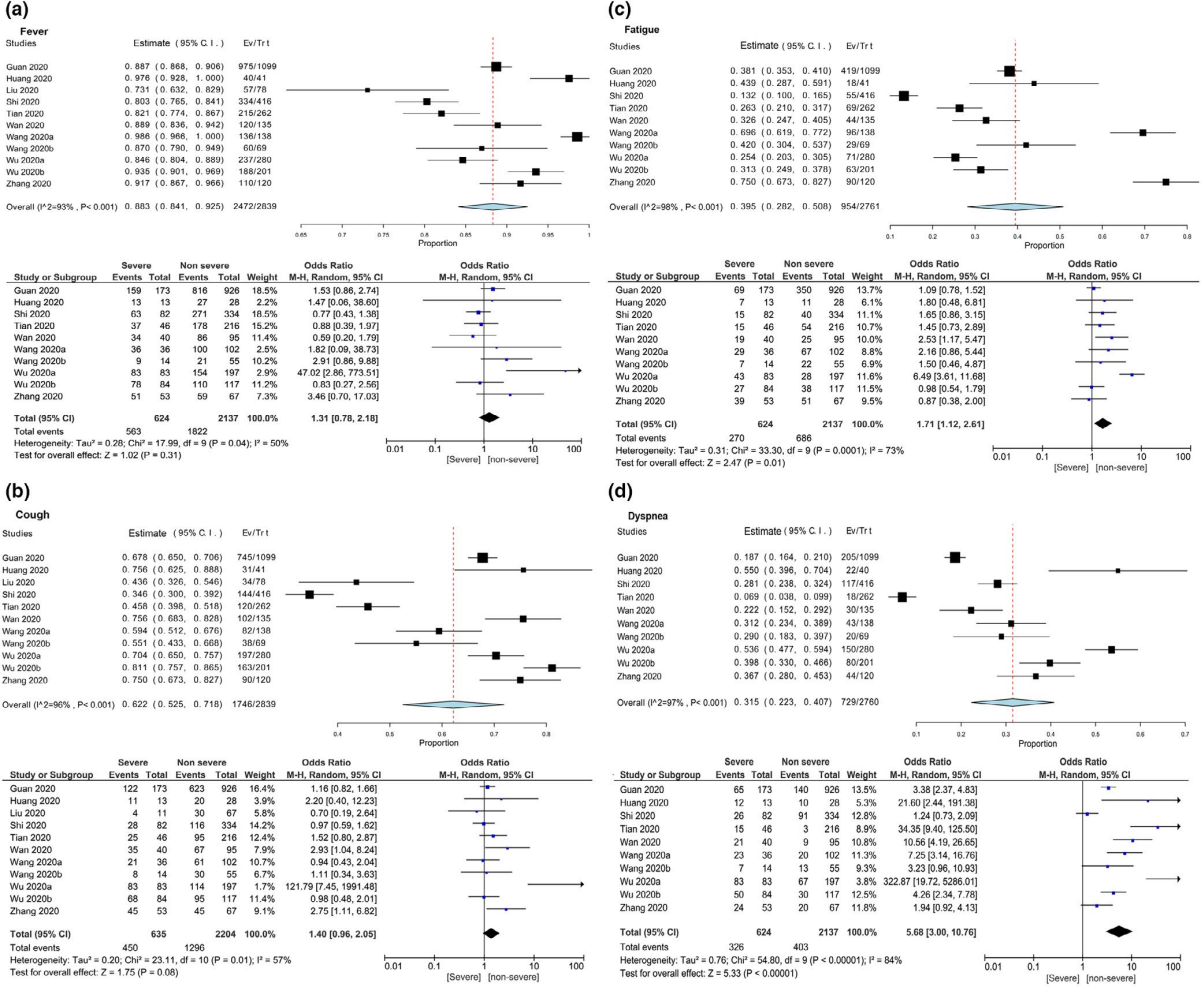
Meta‐analysis for the proportion of fever, cough, fatigue and dyspnoea in COVID‐19 cases. Weights are calculated from binary random‐effects model analysis. Values represent proportions of these clinical features in the COVID‐19 patients and 95% CI. Heterogeneity analysis was carried out using *Q* test, the among studies variation (*I* ^2^ index). Forest plots depict the comparison of the incidences of clinical features in severe and non‐severe patients

We also compared the prevalence of clinical features between severe patients and non‐severe patients. For clinical features, the heterogeneity test results, *I* ^2^ varied from 50%–86%. Thus, the fixed effect model was adopted for further analysis. The result showed that the incidence of fever, cough, sore throat and headache in severe patients were higher than non‐severe group, but without statistical significance [fever: OR = 1.31, 95% CI (0.78–2.18), *Z* = 1.02, *p* = .31; cough: OR = 1.40, 95% CI (0.96–2.05), *Z* = 1.75, *p* = .08; sore throat: OR = 1.68, 95% CI (0.51–5.57), *z* = 0.85, *p* = .39; headache: OR = 1.38, 95% CI (0.59–3.21), *z* = 0.74, *p* = .45] (Table 4; Figure 2). Additionally, the incidence of dyspnoea and fatigue was both statistically significant higher in severe patients compared with the non‐severe patients [dyspnoea: OR = 5.68, 95% CI (3.00–10.76), *Z* = 5.33, *p* < .00001; fatigue: OR = 1.71, 95% CI (1.12–2.61), *Z* = 2.47, *p* = .01] (Figure 2).

**Table 4:** Analysis of severe and non‐severe patients of COVID‐19 by using Mantel–Haenszel test

Variable	Number of studies	OR	95% CI	Severe	Non‐severe	*χ* ^2^_ _ ^a^	*I* ^2^_ _ ^b^	*Z* ^c^	*p*
Male	12	1.28	1.07–1.53	410	1,249	11.16	1	2.76	**.006**
Female	12	0.78	0.65–0.93	282	1,104	11.32	3	2.80	**.005**
Clinical features									
Fever	10	1.31	0.78–2.18	563	1,822	17.99	50	1.02	.31
Cough	11	1.40	0.96–2.05	450	1,296	23.11	57	1.75	.08
Fatigue	10	1.71	1.12–2.61	270	686	33.30	73	2.47	.01
Sore throat	6	1.68	0.51–5.57	65	185	36.33	86	0.85	.39
Dyspnoea	10	5.68	3.00–10.76	326	403	54.80	84	5.33	**<.00001**
Headache	8	1.38	0.59–3.21	77	198	31.56	78	0.75	.45
Comorbidities									
Hypertension	10	3.01	2.03–4.48	202	311	21.07	57	5.45	**<.00001**
Diabetes	10	3.15	2.39–4.15	114	140	17.75	49	8.13	**<.00001**
Cancer	9	3.04	1.81–5.12	28	32	7.79	0	4.19	**<.0001**
COPD	9	6.26	3.34–11.72	26	16	1.53	0	5.72	**<.00001**
Cardiovascular disease	9	5.21	2.69–10.08	110	77	25.34	68	4.91	**<.00001**
Chronic kidney disease	6	3.31	1.73–6.36	20	20	2.66	0	3.60	.0003
Complications									
ARDS	6	17.86	9.21–34.64	158	82	13.34	63	8.53	**<.00001**
Shock	5	29.31	9.66–88.92	27	2	2.22	0	5.97	**<.00001**
Acute kidney injury	6	7.16	2.07–24.69	33	15	12.83	61	3.12	**.002**
Treatments									
Antiviral	9	0.98	0.44–2.20	452	1,323	18.98	68	0.04	.97
Antibiotics	8	6.27	2.80–14.04	485	1,120	18.69	68	4.47	**<.00001**
Glucocorticoids	9	6.36	3.59–11.27	365	527	41.02	80	6.34	**<.00001**
Oxygen support	7	0.21	0.03–1.55	215	919	243.71	98	1.52	.13
CRRT	5	24.79	7.53–81.65	20	1	1.74	0	5.28	**<.00001**
NIV	5	102.30	17.83–587.0	167	9	14.17	72	5.19	**<.00001**
IMV	4	80.70	20.31–320.61	48	0	4.55	34	6.24	**<.00001**

Abbreviations: 95% CI, 95% confidence interval; ARDS, acute respiratory distress syndrome; COPD, chronic obstructive pulmonary disease; CRRT, continuous renal replacement therapy; IMV, invasive mechanical ventilation; NIV, non‐invasive ventilation; OR, odds ratio.

*P*‐values < .05 were considered statistically significant.

^a^ *χ* ^2^ test for heterogeneity.

^b^ *I* ^2^ index to quantify the degree of heterogeneity.

^c^ *Z*‐statistics.

### 3.3 Comorbidities

The most prevalent comorbidities were hypertension (22.6%, 95% CI 16.7%–28.5%), diabetes (11.5%, 95% CI 9.0%–14.1%), cardiovascular disease (10.3%, 95% CI 6.1%–14.5%), followed by cancer (2.5%, 95% CI 1.3%–3.6%), chronic kidney disease (1.8%, 95% CI 0.9%–2.7%) and chronic obstructive pulmonary disease (COPD) (1.6%, 95% CI 0.8%–2.4%). The estimates of comorbidities among identified studies showed significant heterogeneity (Cochran's *Q*) with an *I* ^2^ index varied from 52%–92% (*p* < .05; Table 3).

**Figure 3:**
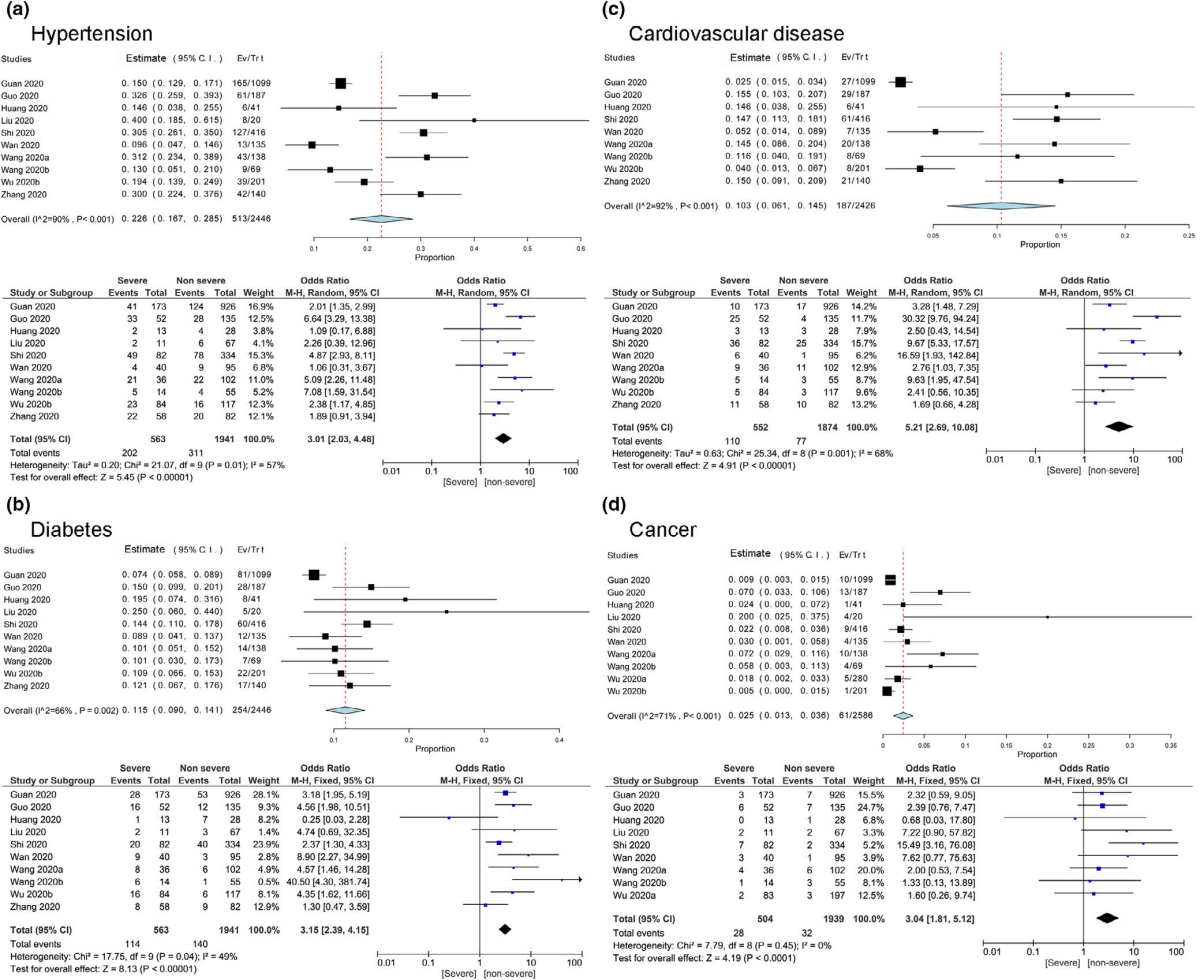
Meta‐analysis for the proportion of hypertension, diabetes, cardiovascular disease and cancer in COVID‐19 cases. Weights are calculated from binary random‐effects model analysis. Values represent proportions of the 4 diseases in the COVID‐19 patients and 95% CI. Heterogeneity analysis was carried out using *Q* test, the among studies variation (*I* ^2^ index). Forest plots depict the comparison of the incidences of the 4 diseases in severe and non‐severe patients

For hypertension, there were 563 cases in severe group (202 hypertension cases, 35.88%) and 1941 cases in non‐severe group (311 hypertension cases, 16.26%). The random effect model was adopted as there was heterogeneity among included studies (*I* ^2^ = 57%). The pooled effect of hypertension in severe and non‐severe cases was 3.01 (OR = 3.01, 95% CI: 2.03–4.48), indicating the incidence of severe risk of the patients with hypertension was 3 times higher than those none (Figure 3). Similarly, the tests result revealed that the incidence of cardiovascular disease in severe group was statistically significant higher than non‐severe group (OR = 5.21, 95% CI (2.69–10.08), *Z* = 4.91, *p* < .00001; Figure 3).

For comorbidities like diabetes, cancer, COPD and chronic kidney disease the heterogeneity test results, *I* ^2^ varied from 0%–49%. So, the fixed effect model was used. Diabetes accounted for 20.25% of severe cases and 7.21% of non‐severe cases. Cancer (5.56% of severe cases vs. 1.65% of non‐severe cases), COPD (5.03% of severe cases vs. 0.84% of non‐severe cases) and chronic kidney disease (4.13% of severe cases vs. 1.14% of non‐severe cases) were also more prevalent in severe patients compared with non‐severe (Table 4).

Furthermore, the incidence of diabetes, cancer, COPD and chronic kidney disease were statistically significant higher in severe patients compared with the non‐severe patients [diabetes: OR = 3.15, 95% CI (2.39–4.15), *Z* = 8.13, *p* < .00001; cancer: OR = 3.04, 95% CI (1.81–5.12), *Z* = 4.19, *p* < .0001; COPD: OR = 6.26, 95% CI (3.34–11.72), *z* = 5.72, *p* < .00001; chronic kidney disease: OR = 3.31, 95% CI (1.73–6.36), *z* = 3.60, *p* = .0003] (Figure 3).

### 3.4 Complications

**Figure 4:**
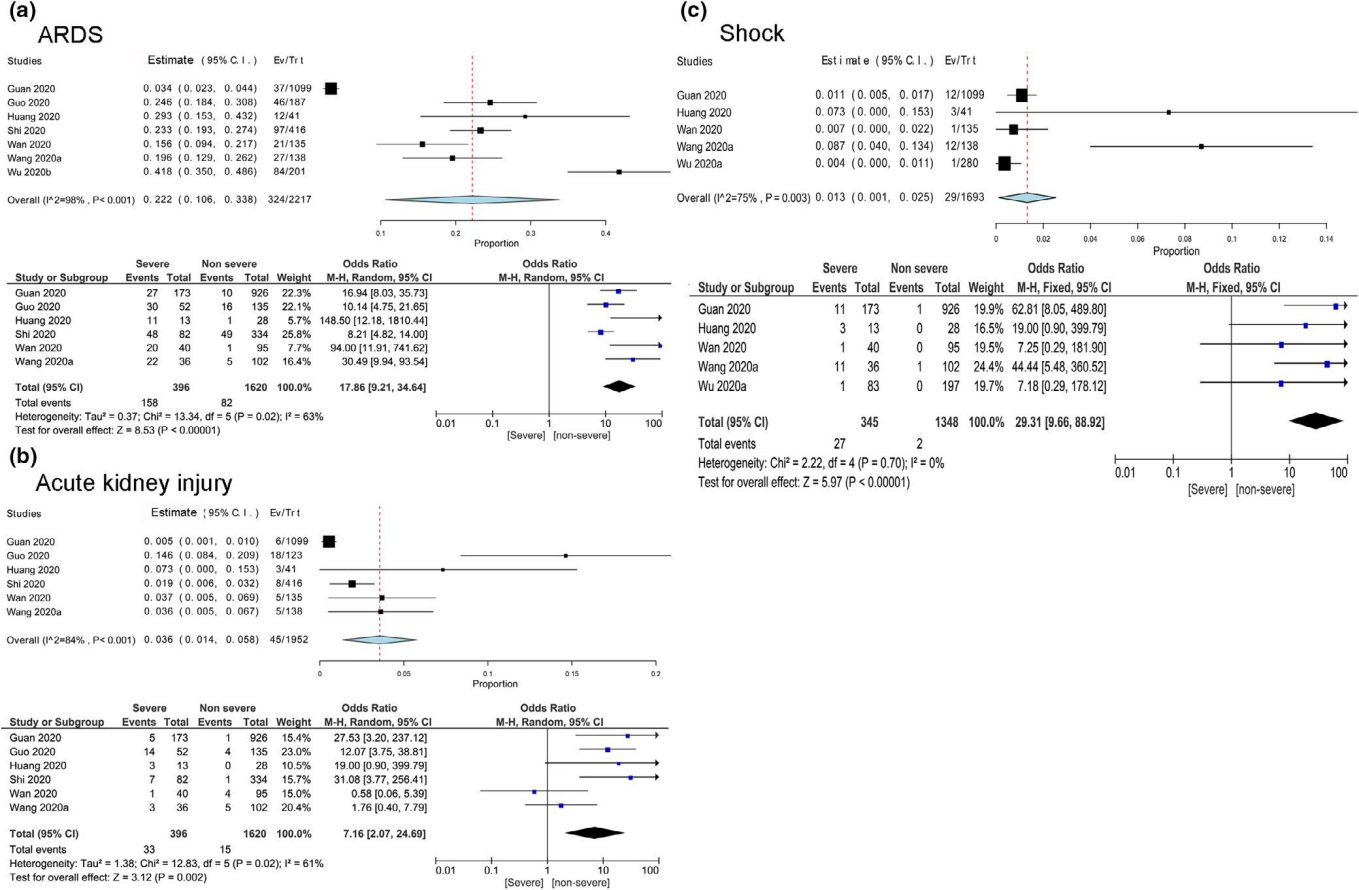
Meta‐analysis for the proportion acute respiratory distress syndrome, acute kidney injury and shock in COVID‐19 cases. Weights are calculated from binary random‐effects model analysis. Values represent proportions of these complications in the COVID‐19 patients and 95% CI. Heterogeneity analysis was carried out using *Q* test, the among studies variation (*I* ^2^ index). Forest plots depict the comparison of the incidences of these three complications in severe and non‐severe patients

Regarding the complications, ARDS (22.2%, 95% CI: 10.6%–33.8%), acute kidney injury (3.6%, 95% CI: 1.4–5.8) and shock (1.3%, 95% CI: 0.1–2.5) were the most prevalent complications (Table 3). The pooled effect of ARDS in severe and non‐severe cases was 17.86 (OR = 17.86, 95% CI: 9.21–34.64), indicating the incidence of severe risk of the patients with ARDS was 17.86 times higher than those none. Similarly, in severe and non‐severe cases the pooled effect of shock and acute kidney injury were 29.31 and 7.16, respectively (Figure 4).

### 3.5 Treatment

With regard to the treatment options, antiviral drugs (87.4%, 95% CI: 78.3–96.4), antibiotics (77.5%, 95% CI: 64.2–90.8), oxygen support (67.6%, 95% CI: 52.0–83.1), glucocorticoids (36%, 95% CI: 20.4–51.6), NIV (24.4%, 95% CI: 10.7–38.0), IMV (8.4%, 95% CI: 4.1–12.6) and CRRT (0.9%, 95% CI: 0.2–1.7) were the most prevalent treatments used to treat COVID‐19 patients (Table 3).

**Figure 5:**
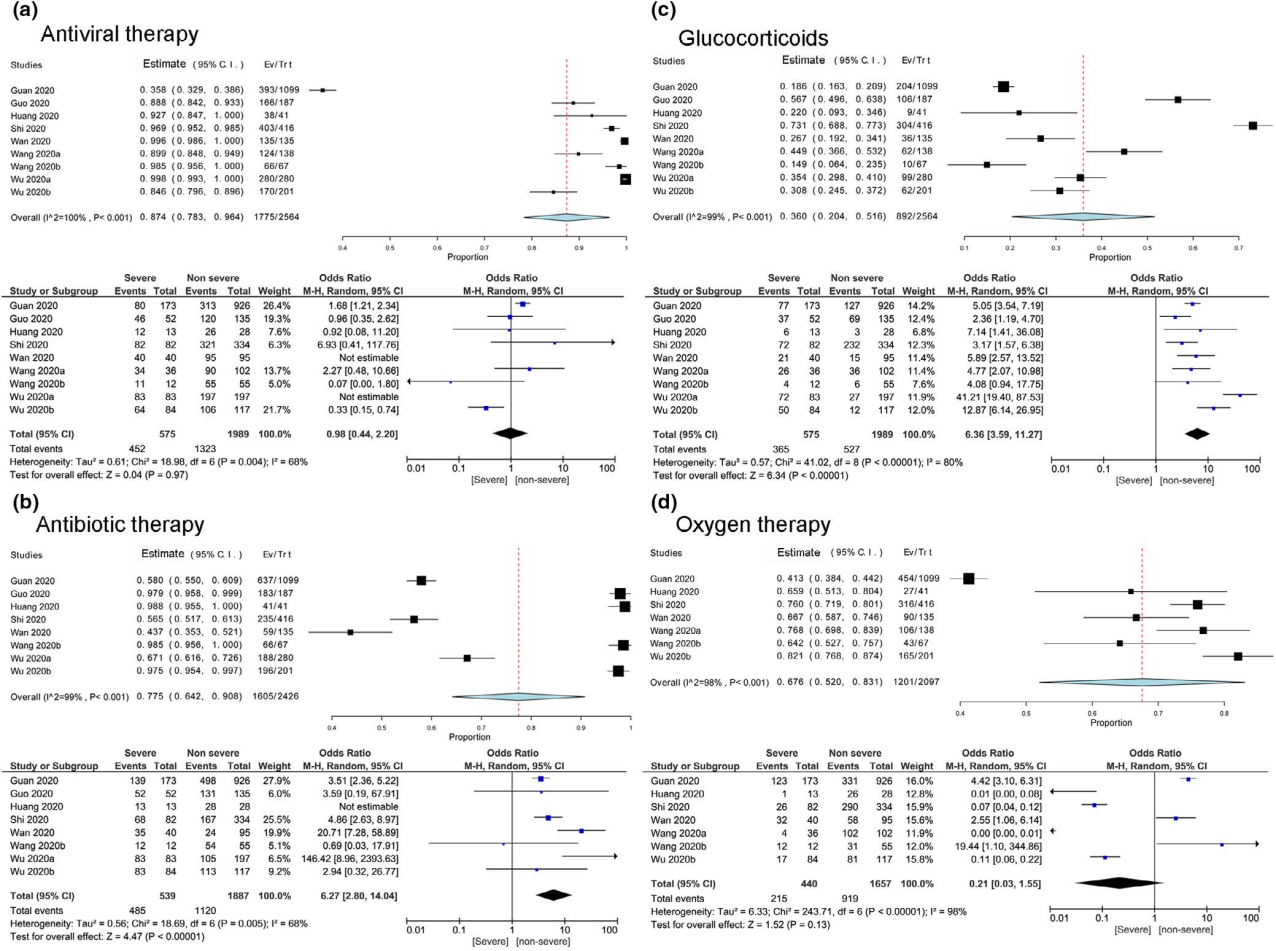
Meta‐analysis for the proportion of antiviral therapy, antibiotic therapy, glucocorticoids and oxygen therapy in COVID‐19 cases. Weights are calculated from binary random‐effects model analysis. Values represent proportions of the 4 therapies in the COVID‐19 patients and 95% CI. Heterogeneity analysis was carried out using *Q* test, the among studies variation (*I* ^2^ index). Forest plots depict the comparison of the incidences of the 4 therapies in severe and non‐severe patients

The frequency of antiviral drugs and oxygen support used in severe cases were higher than the non‐severe cases but without statistical significance [antiviral drugs: OR = 0.98, 95% CI (0.44–2.20), *Z* = 0.04, *p* = .97; oxygen support: OR = 0.21, 95% CI (0.03–1.55), *Z* = 1.52, *p* = .13] (Figure 5). The data also revealed a significant higher incidence of antibiotics, glucocorticoids, IMV, NIV and CRRT use in severe patients compared with the non‐severe patients [antibiotics: OR = 6.27, 95% CI (2.80–14.04), *Z* = 4.47, *p* < .00001; glucocorticoids: OR = 6.36, 95% CI (3.59–11.27), *Z* = 6.34, *p* < .00001; IMV: OR = 80.70, 95% CI (20.31–320.61), *z* = 6.24, *p* < .00001; NIV: OR = 102.30, 95% CI (17.83–587.00), *z* = 5.19, *p* < .00001; CRRT: OR = 24.79, 95% CI (7.53–81.65), *z* = 5.28, *p* < .00001] (Table 4; Figure 5).

## 4 DISCUSSION

The continued occurrence of SARS‐CoV‐2 infection globally is deeply concerning. Despite outbreak prevention and control measures, there is no substantial pandemic change, even after seven months from the onset of SARS‐CoV‐2 outbreak still cases of this infection are increasing at an alarming rate. The present meta‐analysis showed that men were more likely to be infected with COVID‐19 than female counterparts. Previous studies also showed male predominance in incidence of MERS‐CoV and SARS‐CoV infections (Badawi & Ryoo, 2016; Leong et al., 2006). The reason for this might be females are relatively resistant to virus infections as they have stronger innate and adaptive immune responses (Klein & Flanagan, 2016).

The most prevalent symptoms of COVID‐19 were fever, cough, fatigue, dyspnoea and sore throat. The clinical picture of SARS‐CoV‐2 is similar to SARS‐CoV and MERS‐CoV. However, diarrhoea which was prevalent in patients with MERS‐CoV or SARS‐CoV was rare in case of COVID‐19 (Assiri et al., 2013; Fan et al., 2006). Recent study from Singapore and United States of America also revealed that in patients with SARS‐CoV‐2 infection the main symptoms were fever, cough, dyspnoea and sore throat and so on (Arentz et al., 2020; Young et al., 2020). The incidence of fever, cough, dyspnoea, fatigue, sore throat and headache in severe patients was more common than non‐severe group. Previous studies also showed that compared with non‐severe patients, these symptoms were more common in severe patients (Guan, Ni et al., 2020; Huang et al., 2020; Wan et al., 2020; Wang, Hu et al., 2020).

Results of our meta‐analysis demonstrated that the most prevalent comorbidities were hypertension, diabetes and cardiovascular disease. In addition to these comorbidities, cancer, chronic kidney disease and chronic obstructive pulmonary disease (COPD) were also obvious in some patients. The comorbidities identified in our study are in line with previous studies (Guan, Liang et al., 2020; Zumla et al., 2015). A meta‐analysis of 637 MERS‐CoV cases by Badawi et al suggested that hypertension, diabetes and cardiac disease were prevalent in most patients (Badawi & Ryoo, 2016). Our findings suggested that in severe patients of COVID‐19, the incidence of comorbidities such as hypertension, diabetes and cardiac disease, chronic kidney disease etc., was higher than that in non‐severe patients. Hence, severe patients may have adverse clinical outcomes compared with non‐severe cases. In line with our findings, a study by Alqahtani et al. also demonstrated that chronic conditions such as diabetes, hypertension, cardiac disease and renal disease significantly influenced the severity of MERS‐CoV (Alqahtani et al., 2018). Similarly in case of SARS and severe pandemic influenza, comorbidities such as obesity, cardiovascular disease, hypertension, metabolic and dermatologic diseases were strongly associated with the disease severity (Mertz et al., 2013; Moni & Liò, 2014). The down regulation of the host's innate and humoral immune responses by these co‐morbid conditions may limit their ability to counteract any new viral infection. Moreover, severe patients of COVID‐19 had higher concentrations of GCSF, IP10, MCP1, MIP1A and TNFα suggesting that the cytokine storm was associated with disease severity (Huang et al., 2020). Our finding implies that comorbidities should be taken into account when predicting the prognosis in patients with COVID‐19.

With regard to the complication, 22.2% of the patients presented with ARDS, while shock and acute kidney injury were less prevalent. Excessive inflammation reactions with a cytokine storm leading to ARDS were prominently seen in SARS and MERS cases (Kim et al., 2016; Lew et al., 2003). Cytokines including TNFα, IL‐1β, IL‐2, IL‐6, IFNα, IFNβ, IFNγ and MCP‐1 released by cytokine storm induce immune cells to produce free radicals which are major causes of ARDS (Tisoncik et al., 2012). Patients with COVID‐19 pneumonia who had developed ARDS had significantly higher cytokines contributing to cytokine storm (Wu, Chen et al., 2020).

COVID‐19 presents an unprecedented challenge to identify effective drugs for prevention and treatment. Currently, there is not any specific effective antiviral treatment for COVID‐19. Variety of therapies that have been used or proposed for the treatment SARS‐CoV, MERS‐CoV and other viral diseases are also being used for the treatment of COVID‐19 patients. The results of our meta‐analysis revealed that antiviral treatments were administered to 87.4% of the patients. Up to 5%–10% COVID‐19 patients can have severe, potentially life‐threatening course, there have been more than 300 clinical trials going on, and the results are highly anticipated to find an effective antiviral treatment for COVID‐19 (Sanders et al., 2020).

Macrolide antibiotics such as erythromycin and azithromycin have anti‐bacterial, immunomodulatory effects and anti‐inflammatory effects. Although the mechanism of azithromycin against SARS‐CoV‐2 is unclear at present, an open‐label non‐randomized clinical trial by Gautret et al. showed that hydroxychloroquine in combination with azithromycin treatment might be an efficient antiviral therapy for COVID‐19 (Gautret et al., 2020). Our results demonstrated that antibiotics were used in about 77.5% patients and use of antibiotics was significantly higher in severe patients compared with the non‐severe patients. Results of recent studies also showed that antibiotics were predominantly used in COVID‐19 patients (Guan, Ni et al., 2020; Huang et al., 2020; Wan et al., 2020; Wang, Hu et al., 2020).

Previous studies have demonstrated that in patients with SARS and MERS corticosteroids did not improve survival, but resulted in high complications and delayed viral clearance (Arabi et al., 2018; Lee et al., 2004). In our study, glucocorticoid was used in about 36% of patients and it was given to more severe cases. Corticosteroids might have been used to tackle a cytokine storm, to prevent acute lung injury and ARDS (Sanders et al., 2020; Tisoncik et al., 2012). However, due to the limitations of the available literature, the use of glucocorticoids is still controversial. Therefore, the routine use of corticosteroids in patients with COVID‐19 should be avoided unless indicated for another reason. More studies are needed to elucidate the usefulness of corticosteroids in the treatment of COVID‐19.

Our systematic review had limitations. Firstly, most of the data in this study are from retrospective studies and all included studies are from China. Secondly, only few studies are available for inclusion as there are limited studies that compared severe and non‐severe patients; the test efficiency is insufficient. It would be better to include randomized controlled trials in near future from different parts of world with larger sample size to get detailed understanding of COVID‐19.

## 5 CONCLUSION

Based on our results, frontline healthcare professionals such doctors and nurses should be aware that the severe patients might manifest more severe clinical symptoms than the general population. People with pre‐existing comorbidities will need to be considered as a high‐risk group for COVID‐19. Our findings may contribute to a better understanding of patient at risk and can help to improve the assessment and management of severe patients. Furthermore, severity of COVID‐19 has excreted immense pressure on healthcare system of developing countries and early identification of patients at risk for severe illness may reduce the burden on healthcare system.

## CONFLICT OF INTEREST

All authors disclose no conflict of interest.

## AUTHOR CONTRIBUTIONS

Mohan Giri, Anju Puri: Study design. Mohan Giri, Anju Puri, Ting Wang, Shuliang Guo: Data collection and analysis. In addition, all authors read and approved the final manuscript.

## ETHICAL APPROVAL

The study does not require ethical approval because the meta‐analysis is based on published research.

## Data Availability

The data used to support the findings of this study are available from the corresponding author upon request.
